# Perinatal outcomes following maternal pre‐exposure prophylaxis (PrEP) use during pregnancy: results from a large PrEP implementation program in Kenya

**DOI:** 10.1002/jia2.25378

**Published:** 2019-09-09

**Authors:** Julia C Dettinger, John Kinuthia, Jillian Pintye, Felix Abuna, Emily Begnel, Kenneth Mugwanya, Joseph Sila, Harison Lagat, Jared M Baeten, Grace John‐Stewart

**Affiliations:** ^1^ Department of Global Health University of Washington Seattle WA USA; ^2^ Department of Obstetrics/Gynecology Kenyatta National Hospital Nairobi Kenya; ^3^ University of Washington – Kenya Nairobi Kenya; ^4^ Department of Epidemiology University of Washington Seattle WA USA; ^5^ Department of Medicine University of Washington Seattle WA USA; ^6^ Department of Pediatrics University of Washington Seattle WA USA

**Keywords:** pre‐exposure prophylaxis, pregnancy, prevention, children, women, infant, Africa

## Abstract

**Introduction:**

The World Health Organization, while recommending pre‐exposure prophylaxis (PrEP) for HIV‐negative pregnant and postpartum women in HIV high‐burden settings, advocates for continued safety evaluation of PrEP in this population.

**Methods:**

The PrEP Implementation in Young Women and Adolescents (PrIYA) program delivered PrEP to pregnant and postpartum women integrated within routine maternal and child health clinics (MCH) at 16 sites in Western Kenya. PrEP exposure and perinatal outcome data were collected among women obtaining postnatal services during programme evaluation. PrEP use was self‐reported and confirmed with clinical records. Perinatal outcomes including gestational age at birth, birthweight, congenital malformations and infant growth outcomes were abstracted from clinical records for mother‐infant pairs attending the six week visit. Associations between infant outcomes and maternal prenatal PrEP use were assessed using univariate and multivariate logistic and linear regression.

**Results:**

The PrIYA evaluation identified 1530 postpartum mother‐infant pairs with data on prenatal PrEP exposure: 206 with prenatal PrEP use, 1324 without. Median maternal age was 24 years in both groups. PrEP users (any reported PrEP use) were significantly more likely to report HIV risk factors such as: intimate partner violence, sexually transmitted infections and having a partner with positive or unknown HIV status. Most mothers initiated PrEP during the second trimester (n = 116, 57%) and used PrEP for more than one month (n = 110, 58%). The mean birthweight was 3.3 kg and gestational age at birth was 38.5 weeks in both groups. There were no major differences between PrEP exposed and unexposed infants in rates of preterm birth and low birthweight. There were no congenital malformations identified in the PrEP‐exposed group and five reported in the PrEP unexposed group. At six weeks postpartum, infants in both groups had similar growth. No differences in infant outcomes were found by duration PrEP exposure, trimester of PrEP initiation, a subset analysis of women 15 to 24 years old or in multivariate analyses. This analysis demonstrates that monitoring of infant outcomes is feasible within large‐scale programmatic implementation of PrEP among pregnant and postpartum populations.

**Conclusions:**

Pregnancy outcomes and early infant growth did not differ by PrEP exposure.

**Abbreviations**
AGYW: adolescent girls and young women
ART: antiretroviral therapy
CI: confidence interval
HIV: human immunodeficiency virus
IPV: intimate partner violence
IQR: interquartile range
IVD: intravenous drug use
LAZ: length‐for‐age z‐score
LBW: low birthweight
LR: linear regression
MCH: maternal and child health
NASCOP: National AIDS & STI Control Programme
OR: odds ratio
PEP: post‐exposure prophylaxis
PEPFAR: President's Emergency Plan for AIDS Relief
PMTCT: prevention of mother‐to‐child transmission
PNC: postnatal care
PrEP: pre‐exposure prophylaxis
PrIYA: PrEP implementation in young women and adolescents
RAST: risk assessment screening tool
RPR: rapid plasma reagin
STI: sexually transmitted infection
TDF: tenofovir
WAZ: weight‐for‐age z‐score
WHO: World Health Organization
WLZ: weight‐for‐length z‐score

## 1 Introduction

With the implementation of Option B+ for prevention of mother‐to‐child HIV transmission (PMTCT), rates of vertical transmission among women with chronic HIV infections have decreased significantly 1, 2. Current PMTCT strategies are focused on early identification and treatment of women with chronic HIV. With the successes of PMTCT programs in identifying women with chronic HIV infections early in pregnancy, a growing proportion of vertical transmissions are due to acute maternal HIV infection 3, 4. The risk of HIV acquisition during pregnancy and postpartum (among breastfeeding mothers) can be significantly higher than non‐pregnant/postpartum periods 5, 6, and infants born to mothers with acute HIV infection during pregnancy and postpartum are at high risk of acquiring HIV 3, 4, 5, 7, 8. Pre‐exposure prophylaxis (PrEP) is an effective HIV prevention option for mothers at risk of acquiring HIV during pregnancy and postpartum 9, 10. As PrEP implementation expands, it is critical to monitor PrEP use during pregnancy and maternal/infant outcomes 11, 12, 13.

In PrEP efficacy trials, pregnant women were excluded from the studies and PrEP was discontinued at diagnosis of pregnancy resulting in limited data regarding the impact of PrEP use in pregnancy 14, 15. Data from these efficacy trials and from subsequent PrEP demonstration projects that allowed women to continue using PrEP provide evidence that PrEP is safe for use during pregnancy 14, 15, 16. Additionally, there is a body of evidence for the safety of tenofovir (TDF)‐based antiretroviral therapy (ART) regimens among HIV‐infected mothers 10, 17, 18, 19, 20. TDF is the primary active ingredient in PrEP. A systematic review and meta‐analysis found no statistically significant differences in stillbirth/miscarriage, birthweight, preterm birth, congenital malformations or infant or maternal mortality among women using a TDF‐based ART compared with other ART regimens or no exposure 19. Most studies have shown no statistical difference on infant growth z‐scores by TDF exposure 10; two studies showed conflicting results regarding the effect of TDF‐ART exposure on one‐year length‐for‐age z‐scores 18, 21. Given this evidence, the World Health Organization (WHO) and many countries, including Kenya, recommend PrEP for use during pregnancy and breastfeeding for women at risk of acquiring HIV 22, 23, 24, 25, 26. However, WHO recommends continued research and monitoring to assess the impact of PrEP exposure *in utero* or during breastfeeding on infant outcomes 22.

The PrEP Implementation in Young Women and Adolescents (PrIYA) program integrates PrEP delivery within routine maternal child health (MCH) and family planning (FP) clinics in Kisumu County, Kenya. As part of an evaluation of PrIYA, women attending infant postnatal visits were assessed for previous receipt of PrEP within MCH clinics and data from their clinical records were abstracted. With data collected from clinical records as part of the PrIYA evaluation, we were able to respond to the WHO call for continued surveillance of infant outcomes. Our objective was to determine if PrEP exposure in pregnancy was associated with any adverse birth outcomes. Thus, we compared birth outcomes among women with a history of PrEP use in pregnancy to those without.

## 2 Methods

### 2.1 Overview of PrIYA program

PrIYA, implemented by the University of Washington in collaboration with the Kisumu County, Department of Health, was a Dreams Innovation Challenge program funded by PEPFAR and managed by JSI Research & Training Institute, Inc. Between June 2017 and June 2018, PrIYA nurses screened all women attending MCH and FP clinics at 16 facilities in Kisumu County for PrEP, using Kenyan Ministry of Health (NASCOP) guidelines 22. Any woman who wanted to initiate PrEP after standard counselling was able to initiate PrEP. In addition, women were screened for HIV risk factors using a Risk Assessment Screening Tool (RAST) that was developed from a combination of a validated risk assessment tool and the Kenyan Ministry of Health (NASCOP) risk guidelines 23, 27. Specifically, a participant was identified as at risk, and behaviourally eligible for PrEP, if they had >6 lifetime sexual partners, reported a partner of unknown or known positive HIV status, had positive syphilis test results or in the last six months reported any of the following: sex without a condom, transactional sex, diagnosis or treatment for an sexually transmitted infection (STI), forced to have sex, experienced intimate partner violence (IPV), shared needles during intravenous drug use or used PEP more than twice. The RAST tool also collected basic demographic information (age, marital status and gestational age).

Women who expressed interest in PrEP or screened as high risk using the RAST were counselled on PrEP, underwent point‐of‐care creatinine clearance testing for medical eligibility (StatSensor^®^ Point‐of‐Care Creatinine and eGFR Analyzers, Nova Biomedical) and were prescribed PrEP and followed up according to NASCOP guidelines 23, 28. For all women who underwent medical eligibility screening for PrEP, a NASCOP PrEP card was completed as part of PrEP delivery. Women prescribed PrEP were initially given a one‐month prescription; however, after the first follow‐up visit prescribers could give up to three months of PrEP. Data from the RAST tool, PrEP enrolment and PrEP follow‐up cards were abstracted. PrEP discontinuation was ascertained at a follow‐up clinic visit or via a phone call with PrIYA nurses.

### 2.2 PrIYA program evaluation

PrIYA began a programme evaluation in June 2018 aimed at understanding the penetration of PrEP screening in MCH clinics. All mother and infant pairs attending postnatal care (PNC) visits at PrIYA MCH clinic sites between June 2018 and November 2018 were assessed for whether they had been screened previously for HIV risk by PrIYA nurses, including self‐report and/or presence of a screening ID number or PrEP refill visit dates in the MCH card. If they reported PrEP screening, information was collected on whether they had taken PrEP during pregnancy or postpartum. Women whose prenatal PrEP screening was confirmed by clinical records from the time of PrEP screening/initiation were included in this retrospective cohort. The data collection period covered the period between initial antenatal PrEP screening/initiation and data abstraction at the six week postpartum clinic visit. The gestational age at PrEP screening/initiation varied substantially as women attending antenatal care at any gestational age were eligible for PrEP screening and initiation. Data on gestational age at birth, birthweight, congenital malformations and six week infant growth measures were abstracted from the clients’ MCH booklet. The MCH booklet is a Kenya Ministry of Health medical record completed by MCH nurses at specific prenatal and postnatal milestones. All data were abstracted and managed using the mobile REDCap (Research Electronic Data Capture) tools hosted at the University of Washington Institute for Translational Health Science 29.

### 2.3 PrEP exposure and outcome definitions

PrEP use/non‐use was as reported by mothers including review of MCH cards for PrEP screening ID numbers or PrEP refill visit information, and confirmed by clinic records from the time of PrEP screening. Women whose pregnancy PrEP use or non‐use could not be confirmed in their medical records were excluded from all analyses. Gestational age at PrEP initiation was calculated using the gestational age reported on the PrEP card, and trimester of PrEP initiation was defined based on the gestational age at the time of PrEP prescription. All PrEP exposed participants in this evaluation started PrEP during pregnancy; there was no reported preconception PrEP exposure.

If date of PrEP initiation and discontinuation were available from PrEP clinical records, duration of PrEP use was calculated as the number of days between initiation and discontinuation. Length of prenatal PrEP exposure was defined as short, <1 month, operationally defined as <45 day to account for challenges with timing of PrEP refills or long, ≥1 month operationally defined as ≥45 days, based on date of PrEP discontinuation or date of birth, whichever was earlier. If the date of discontinuation was not available in PrEP clinical records, women were asked about the duration of their PrEP use. Women who reported PrEP use of <1 month were included in the short duration of PrEP use category, and women who reported ≥1 month PrEP use were categorized as long PrEP duration. Mothers who reported receiving a PrEP prescription but never swallowed the pills were excluded from the analysis as were mothers who used PrEP in the postpartum period only. Measures of PrEP exposure were based on clinical records and self‐report, no other markers (biomarkers, pill counts, MEMS, etc.) were used.

Gestational age at birth was abstracted from the MCH card, and if this was missing from the MCH card, was calculated based on the gestational age at the time of PrEP screening (RAST card) and the date of birth. Preterm birth was defined as gestational age at birth <37 weeks 30. Birthweight reported on the MCH card was used to calculate low birthweight (LBW) (defined as birthweight <2500 g among infants born ≥37 weeks gestational age). Infant growth outcomes at six weeks postpartum were calculated using weight‐for‐age (WAZ), length‐for‐age (LAZ) and weight‐for‐length (WLZ) z‐scores using the WHO standards 31. Underweight, stunting, and wasting were defined as a WAZ, LAZ and WLZ z‐scores ≤−2. Since information was abstracted from the clinical records of mother‐infant pairs attending the six week postpartum visit, we were unable to collect information on loss of pregnancy or early infant deaths.

### 2.4 Statistical analyses

Descriptive statistics were calculated for demographic and HIV‐risk profiles using data collected from the RAST card and compared between PrEP exposed and unexposed women. Logistic regression models, using the cluster function for clinic, were used to estimate the odds of binary birth and infant growth outcomes by prenatal PrEP exposure. Linear regression models, using the cluster function for clinic, were used to estimate the regression coefficients for continuous infant growth outcomes by prenatal PrEP exposure. To adjust for differences between PrEP exposed and unexposed groups, partner HIV status, dichotomized as positive/unknown (women with partners of unknown or positive status were classified high risk) and negative, and gestational age at the time of PrEP screening were included in the primary multivariate analysis. For the secondary analysis assessing the impact of trimester of PrEP initiation and duration of PrEP exposure, partner status was included in the multivariate models. A subset analysis of outcomes of adolescent girls and young women (AGYW) aged 15 to 24 was also conducted. Statistical analyses were performed with STATA 14.0/IC (Stata corporation, College Station, TX).

### 2.5 Human subjects

The PrIYA program was implemented following the Kenyan NASCOP guidelines, which include a behavioural risk assessment. The risk assessment in the national programme includes obtaining sensitive information about HIV risk factors. All participants in the PrIYA program provided oral consent prior to participation in the PrIYA program. The PrIYA program evaluation protocol was reviewed by both University of Washington IRB and Kenyatta National Hospital Ethical Review Committee (ERC) and approval for oral consent (to avoid programmatic disruption that could occur with written consent) was obtained from both IRB and ERC for the PrIYA program evaluation. All women provided oral consent for the abstraction of their clinical records.

## 3 Results

**Figure 1:**
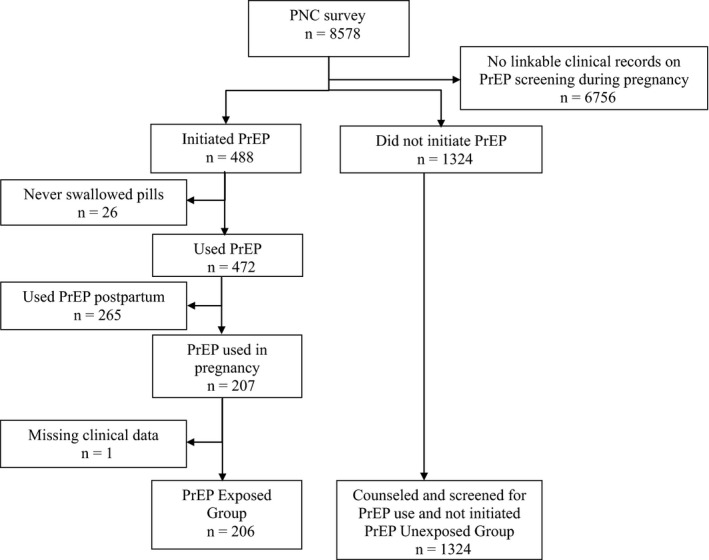
Flow diagram of inclusion in the infant outcomes analysis

Overall, 8578 women and infant pairs were approached to participate in the PrIYA evaluation while receiving postnatal care, of whom 1530 (18%) women had PrEP screening records available. Among these 1530 women, 206 reported prenatal PrEP use and 1324 reported no prenatal PrEP use (Figure 1). The median age was 24 years, and did not differ between women who did and did not use PrEP (Table 1). The majority of women were married (85%); 1% reported having a partner who was HIV positive and 30% of women reported having a partner of unknown HIV status. The median gestational age at the time of antenatal PrEP screening was 27 weeks (Interquartile Range (IQR): 22, 32) and median gestational age at birth was 38.5 weeks (IQR: 38.5, 38.6) with 6% of all infants born preterm. The mean weight for infants at six weeks postpartum was 5.1 kg (95% Confidence Interval (CI): 5.05 to 5.1), and the prevalence of underweight (3%), stunting (9%) and wasting (7%) at six weeks were low.

**Table 1:** Characteristics of women and infants stratified by history of PrEP Exposure

Demographic characteristics	n (%) or median (IQR)		
	Overall (N = 1530)	PrEP exposed (N = 206)	PrEP unexposed (N = 1324)
Age (years)	24.0 (21.0 to 28.0)	24.0 (20.0 to 27.0)	24.0 (21.0 to 28.0)
Age category (years)			
<18	64 (4.2)	11 (5.3)	53 (4.0)
18 to 24	739 (48.3)	102 (49.5)	637 (48.1)
25 to 29	387 (25.3)	54 (26.2)	333 (25.2)
30 to 34	206 (13.5)	22 (10.7)	184 (13.9)
≥35	73 (4.8)	15 (7.3)	58 (4.4)
Missing	61 (4.0)	2 (1.0)	59 (4.5)
Married	1305 (85.3)	174 (84.5)	1131 (85.4)
Gestational age at PrEP screening^a^, ^b^	27.0 (22.0 to 32.0)	25.0 (20.0 to 30.0)	28.0 (24.0 to 32.0)
Behavioural risk factors			
HIV status of sexual partner(s)^b^			
Negative	978 (63.9)	88 (42.7)	890 (67.2)
Unknown	459 (30.0)	102 (49.5)	357 (27.0)
Positive	21 (1.4)	14 (6.8)	7 (0.5)
Missing	72 (4.7)	2 (1.0)	70 (5.3)
RPR results^a^, ^b^			
Reactive	4 (0.5)	2 (1.1)	2 (0.3)
Nonreactive	632 (77.3)	109 (59.6)	520 (82.4)
Not done/unknown	157 (19.3)	58 (31.7)	99 (15.7)
Missing	24 (2.9)	14 (7.7)	10 (1.6)
HIV tested as couple during ANC^a^	46 (5.7)	7 (3.8)	39 (6.2)
Missing	24 (2.9)	15 (8.2)	9 (1.4)
Behavioural risk factors (last six months)			
Ever had sex without a condom^b^	1364 (92.9)	199 (96.6)	1165 (88.0)
Engaged in sex in exchange of money/favours	2 (0.1)	1 (0.5)	1 (0.1)
Diagnosed with or treated for an STI	4 (0.3)	3 (1.5)	1 (0.1)
Forced to have sex	6 (0.4)	1 (0.5)	5 (0.4)
Experienced IPV^b^	27 (1.8)	12 (5.8)	15 (1.1)
Shared needles while engaging in IVD	0 (0.0)	0 (0.0)	0 (0.0)
Used PEP >2 times	0 (0.0)	0 (0.0)	0 (0.0)

a Data collected only for clients who were linked to their PrEP screening data and were pregnant at the time of PrEP screening (N = 814)

b *p *< 0.05.

Mothers who used PrEP were screened by PrIYA nurses at an earlier gestational age (25 weeks, IQR: 20 to 30) than mothers who did not use PrEP (28 weeks, IQR: 24 to 32). Mothers who used PrEP were more likely to report HIV risk factors including: a partner of unknown or HIV‐positive status (56% vs. 27%), sex without a condom in the past six months (97% vs. 92%), experienced intimate partner violence (6% vs. 1%) and a reactive syphilis test result (1% vs. 0.3%) (Table 1).

The mean gestational age at birth was 38.5 weeks in both PrEP exposed (95% CI: 38.3 to 38.7) and unexposed infants (95% CI: 38.5, 38.6). PrEP exposed infants had lower rates of preterm birth (3%, 95% CI: 1.9, 6.2 vs. 7%, 95% CI: 2.9, 15.6), and LBW (0.5%, 95% CI: 0.1, 4.7 vs. 2%, 95% CI: 1.6, 3.7); however, lack of precision in these estimates limit our ability to interpret these results (Table 2). There were five reports of congenital malformation in the PrEP unexposed group and none in the PrEP exposed group. At six weeks postpartum, infants in both groups had similar infant growth outcomes. The mean weight for PrEP exposed infants was 5.0 kg (95% CI: 4.9, 5.1) and in PrEP unexposed infants was 5.1 kg (95% CI: 5.0, 5.1). Infant weight (WAZ: 0.4, 95% CI: 0.2, 0.6), length (LAZ: 0.1, 95% CI: −0.1, 0.4) and weight‐for‐length (WHZ: 0.6, 95% CI: 0.4, 0.9) z‐scores were close to normal values among PrEP exposed infants and did not differ from PrEP unexposed infants. There were no differences in the proportion of infants categorized as underweight (3%, 95% CI: 1.1, 6.5 vs. 3%, 95% CI: 1.4, 4.9), stunted (8%, 95% CI: 4.1, 13.6 vs. 9%, 95% CI: 6.3, 12.3) or wasted (6%, 95% CI: 2.4, 15.8 vs. 8%, 95% CI: 4.8, 11.6) between PrEP exposed and unexposed infants. Findings were similar in multivariate analyses adjusted for baseline differences between PrEP exposed and unexposed mothers, specifically partner HIV status and gestational age at PrEP screening (Table 2), and in a subset analysis of AGYW mothers (Table S1).

**Table 2:** Birth and infant growth outcomes by prenatal PrEP exposure

	Overall (N = 1530)		PrEP exposed (N = 206)		PrEP unexposed (N = 1324)		Unadjusted coeff. or OR (95% CI)	Adjusted coeff. or OR (95% CI)
	Mean or N (%)	95% CI	Mean or N (%)	95% CI	Mean or N (%)	95% CI		
Birth outcomes								
Gestational age at birth (weeks)^a^	38.5	38.3, 38.8	38.5	38.0, 39.0	38.6	38.3, 38.8	−0.05 (−0.49, 0.39)	0.0055 (−0.38, 0.39)
Preterm birth^b^	95 (6.4)	2.9, 13.6	7 (3.4)	1.9, 6.2	88 (6.9)	2.9, 15.6	0.48 (0.17, 1.38)	0.43 (0.15, 1.21)
Congenital malformation^b^	5 (0.3)	0.1, 1.3	0 (0.0)	–	5 (0.4)	0.1, 1.5	–	–
Birthweight (kg)^a^	3.3	3.3, 3.4	3.4	3.3, 3.5	3.3	3.3, 3.3	0.06 (−0.05, 0.16)	0.10* (0.0055, 0.20)
Low birthweight^b^	30 (2.2)	1.4, 3.3	1 (0.5)	0.1, 4.7	29 (2.5)	1.6, 3.7	0.73 (0.31, 1.73)	0.58 (0.20, 1.73)
Six week infant growth outcomes								
Weight (kg)^a^	5.1	4.9, 5.2	5.0	4.9, 5.2	5.1	4.9, 5.2	−0.04 (−0.16, 0.07)	−0.022 (−0.19, 0.14)
Absolute weight‐for‐age z‐score (WAZ)^a^	0.4	0.2, 0.6	0.4	0.1, 0.6	0.4	0.2, 0.6	−0.03 (−0.21, 0.14)	0.022 (−0.18, 0.23)
Moderate‐to‐severe underweight^b^	31 (2.6)	1.5, 4.7	4 (2.7)	1.1, 6.5	27 (2.6)	1.4, 4.9	1.07 (0.43, 2.66)	0.64 (0.17, 2.35)
Length (cm)^a^	55.8	55.1, 56.5	55.8	54.8, 56.8	55.8	55.1, 56.5	−0.03 (−0.93, 0.87)	−0.27 (−1.37, 0.82)
Absolute length‐for‐age z‐score (LAZ)^a^	0.1	−0.3, 0.4	0.1	−0.3, 0.6	0.1	−0.3, 0.4	0.05 (−0.35, 0.45)	−0.086 (−0.60, 0.43)
Moderate‐to‐severe stunting^b^	104 (8.7)	6.4, 11.8	11 (7.6)	4.1, 13.6	93 (8.9)	6.3, 12.3	0.84 (0.40, 1.75)	1.38 (0.49, 3.92)
Weight‐for‐length (WHZ) Z‐score^a^	0.6	0.3, 0.9	0.6	0.1, 1.2	0.6	0.2, 0.9	0.04 (−0.38, 0.46)	0.21 (−0.18, 0.61)
Moderate‐to‐severe wasting^b^	88 (73)	4.7, 11.4	9 (6.3)	2.4, 15.8	79 (7.5)	4.8, 11.6	0.81 (0.36, 1.81)	0.75 (0.26, 2.18)

Adjusted for gestational age at PrEP screening and Partner HIV status.

^a^Coefficient; ^b^odds ratio.

*p < 0.05.

Most mothers who used PrEP in pregnancy initiated PrEP during the second trimester (n = 116, 57%), followed by third trimester PrEP initiation (n = 77, 38%). Outcomes, including gestational age at birth, preterm birth and six week infant growth were similar between mothers who initiated PrEP in the first, second or third trimesters and PrEP unexposed infants (Table 3). When stratified by duration of PrEP exposure, most mothers used PrEP for more than 45 days (n = 110, 58%). The median gestational age at birth was 38.5 weeks in all three groups: PrEP unexposed, short PrEP duration and long PrEP duration. Women (n = 17) were excluded from the PrEP duration analysis if they reported PrEP initiation (confirmed with PrEP prescription), but did have refill or self‐reported PrEP duration data available. No differences were found in comparisons of the short or long PrEP duration groups to the PrEP unexposed group for low birthweight or six week growth outcomes (Table 4). Multivariate analyses adjusting for partner HIV status did not significantly impact these results (Tables 3,4). The subset analysis among AGYW found similar results (Tables S2 and S3).

**Table 3:** Birth and infant growth outcomes by trimester of PrEP initiation

	Unexposed		Trimester of PrEP initiation^a^											
	Reference (n = 1324)		First (n = 12)				Second (n = 116)				Third (n = 77)			
	Mean N (%)	95% CI	Mean N (%)	95% CI	Coeff. or OR (95% CI)	Adjusted coeff. or OR ( 95% CI)	Mean N (%)	95% CI	Coeff. or OR (95% CI)	Adjusted coeff. or OR (95% CI)	Mean N (%)	95% CI	Coeff. or OR (95% CI)	Adjusted coeff. or OR (95% CI)
Birth outcomes														
Gestational age at birth (weeks)^b^	38.6	38.3, 38.8	38.5	37.5, 39.5	−0.04 (−0.72, 0.63)	−0.11 (−0.78, 0.56)	38.4	37.9, 38.9	−0.13 (−0.56, 0.31)	−0.20 (−0.60, 0.20)	38.7	38.1, 39.4	0.17 (−0.42, 0.77)	0.13 (−0.45, 0.70)
Preterm birth^b^	88 (6.9)	2.9, 15.6	1 (8.3)	0.9, 48.9	1.22 (0.13, 11.75)	1.52 (0.17, 13.4)	5 (4.5)	2.1, 9.3	0.62 (0.20, 1.94)	0.71 (0.25, 1.96)	0 (0.0)	–	–	–
Congenital malformation^c^	5 (0.4)	0.1, 1.5	0 (0.0)	–	–	–	0 (0.0)	–	–	–	0 (0.0)	–	–	–
Birthweight (kg)^b^	3.3	3.3, 3.3	3.2	2.9, 3.4	−0.15 (−0.35, 0.046)	−0.14 (−0.35, 0.061)	3.3	3.2, 3.4	0.02 (−0.10, 0.14)	0.017 (−0.10, 0.13)	3.5	3.3, 3.7	0.17 (−0.03, 0.36)	0.17 (−0.017, 0.35)
Low birthweight (<2.5 kg)^c^^,^ d	29 (2.5)	1.6, 3.7	0 (0.0)	–	–	–	1 (0.9)	0.1, 8.3	0.37 (0.045, 3.03)	0.38 (0.048, 3.06)	0 (0.0)	–	–	–
Infant growth outcomes at six weeks postpartum														
Weight (kg)^b^	5.1	4.9, 5.2	4.8	4.2, 5.5	−0.25 (−0.80, 0.30)	−0.22 (−0.76, 0.33)	4.9	4.7, 5.1	−0.14 (−0.31, 0.04)	−0.12 (−0.30, 0.058)	5.2	4.9, 5.4	0.11 (−0.11, 0.33)	0.13 (−0.099, 0.35)
Absolute WAZ^b^	0.4	0.2, 0.6	−0.04	−1.2, 1.2	−0.45 (−1.37, 0.18)	−0.39 (−1.32, 0.54)	0.3	−0.1, 0.7	−0.13 (−0.43, 0.18)	−0.096 (−0.41, 0.22)	0.5	0.2, 0.9	0.13 (−0.18, 0.45)	0.17 (−0.15, 0.48)
Moderate–to–severe underweight^c^	27 (2.6)	1.4, 4.9	0 (0.0)	–	–	–	3 (3.7)	1.4, 9.4	1.46 (0.61, 3.47)	1.24 (0.62, 2.48)	1 (1.7)	0.3, 9.2	0.66 (0.11, 3.93)	0.54 (0.089, 3.24)
Length (cm)^b^	55.8	55.1, 56.5	58.2	54.9, 61.6	2.39 (−1.42, 6.20)	2.51 (−1.54, 6.56)	55.4	54.4, 56.4	−0.42 (−1.17, 0.33)	−0.37 (−1.24, 0.51)	56.1	54.7, 57.5	0.24 (−0.84, 1.33)	0.31 (−0.91, 1.53)
Absolute LAZ^b^	0.1	−0.3, 0.4	1.2	−0.6, 2.9	1.11 (−0.91, 3.13)	1.17 (−0.98, 3.32)	−0.02	−0.5, 0.4	−0.09 (−0.42, 0.24)	−0.062 (−0.44, 0.32)	0.2	−0.5, 0.9	0.13 (−0.41, 0.67)	0.16 (−0.46, 0.77)
Moderate–to–severe stunting^c^	93 (8.9)	6.3, 12.3	0 (0.0)	–	–	–	8 (9.9)	4.6, 19.9	1.11 (0.46, 2.67)	1.07 (0.43, 2.67)	3 (5.3)	2.2, 12.2	0.57 (0.24, 1.34)	0.54 (0.22, 1.30)
Weight–for–length (WHZ) z–score^b^	0.6	0.2, 0.9	0.2	−3.1, 3.4	−0.43 (−2.68, 1.83)	−0.38 (−2.70, 1.93)	0.6	0.04, 1.2	0.011 (−0.48, 0.51)	0.037 (−0.54, 0.61)	0.7	0.2, 1.3	0.12 (−0.29, 0.54)	0.15 (−0.35, 0.65)
Moderate–to–severe wasting^c^	79 (7.5)	4.8, 11.6	1 (20.0)	0.8, 88.9	3.03 (0.33, 27.7)	3.37 (0.31, 36.25)	5 (6.2)	2.0, 17.6	0.79 (0.27, 2.33)	0.85 (0.28, 2.60)	3 (5.3)	0.9, 24.5	0.67 (0.18, 2.53)	0.73 (0.18, 3.00)

Adjusted for partner HIV status.

^a^Participant without trimester of PrEP initiation data excluded from analysis; ^b^coefficient; ^c^odds ratio; ^d^infants born before 37 weeks gestational age were excluded from this analysis.

*p < 0.05.

**Table 4:** Birth and infant growth outcomes by duration of PrEP use

	Unexposed		PrEP duration^a^							
	Reference (n = 1324)		Short duration (n = 79)				Long duration (n = 110)			
	Mean N (%)	95% CI	Mean N (%)	95% CI	Coeff. or OR (95% CI)	Adjusted coeff. or OR (95% CI)	Mean N (%)	95% CI	Coeff. or OR (95% CI)	Adjusted coeff. or OR (95% CI)
Birth outcomes										
Gestational age at birth (weeks)^b^	38.6	38.3, 38.8	38.5	37.9, 39.0	−0.079 (−0.62, 0.46)	−0.12 (−0.64, 0.40)	38.5	37.9, 39.1	−0.04 (−0.53, 0.45)	−0.13 (−0.57, 0.31)
Preterm birth^c^	88 (6.9)	2.9, 15.6	2 (2.6)	1.0, 6.6	0.35 (0.089, 1.40)	0.39 (0.11, 1.39)	4 (3.7)	1.5, 9.0	0.51 (0.16, 1.66)	0.61 (0.21, 1.72)
Congenital malformation^c^	5 (0.4)	0.1, 1.5	0 (0.0)				0 (0.0)			
Birthweight (kg)^b^	3.3	3.3, 3.3	3.4	3.3, 3.6	0.13 (−0.019, 0.29)	0.13 (−0.018, 0.28)	3.3	3.2, 3.4	−0.011 (−0.10, 0.081)	−0.012 (−0.10, 0.076)
Low birthweight (<2.5 kg)^c^^,^ d	29 (2.5)	1.6, 3.7	0 (0.0)	–	–	–	1 (1.0)	0.1, 8.9	0.38 (0.046, 3.09)	0.40 (0.050, 3.20)
Infant growth outcomes at six weeks postpartum										
Weight (kg)^b^	5.1	4.9, 5.2	5.1	4.9, 5.3	0.050 (−0.097. 0.20)	0.062 (−0.10, 0.22)	4.9	4.7, 5.1	−0.14 (−0.30, 0.011)	−0.13 (−0.28, 0.026)
Absolute WAZ^b^	0.4	0.2, 0.6	0.5	0.2, 0.8	0.11 (−0.10, 0.32)	0.14 (−0.096, 0.37)	0.2	−0.03, 0.5	−0.17 (−0.41, 0.07)	−0.13 (−0.37, 0.11)
Moderate–to–severe underweight^c^	27 (2.6)	1.4, 4.9	0 (0.0)	–	–	–	4 (5.1)	2.2, 11.0	2.04 (0.85, 4.90)	1.67 (0.79, 3.53)
Length (cm)^b^	55.8	55.1, 56.5	56.1	54.7, 57.4	0.24 (−0.78, 1.26)	0.29 (−0.87, 1.46)	55.7	54.7, 56.6	−0.15 (−1.13, 0.83)	−0.085 (−1.25, 1.08)
Absolute LAZ^b^	0.1	−0.3, 0.4	0.2	−0.5, 0.9	0.10 (−0.41, 0.61)	0.13 (−0.47, 0.72)	0.1	−0.3, 0.6	0.076 (−0.37, 0.52)	0.12 (−0.42, 0.66)
Moderate–to–severe stunting^c^	93 (8.9)	6.3, 12.3	4 (7.1)	2.8, 16.9	0.79 (0.30, 2.11)	0.74 (0.26, 2.09)	5 (6.4)	3.2, 12.2	0.69 (0.31, 1.55)	0.64 (0.28, 1.49)
Weight–for–length (WHZ) z–score^c^	0.6	0.2, 0.9	0.6	−0.1, 1.4	0.049 (−0.51, 0.61)	0.067 (−0.57, 0.70)	0.5	−0.1, 1.1	−0.063 (−0.56, 0.43)	−0.038 (−0.64, 0.56)
Moderate–to–severe wasting^c^	79 (7.5)	4.8, 11.6	4 (7.3)	2.1, 22.6	0.95 (0.37, 2.44)	1.02 (0.36, 2.93)	5 (6.5)	2.0, 18.8	0.83 (0.29, 2.41)	0.92 (0.29, 2.88)

Adjusted for partner HIV status.

^a^17 participants without information on PrEP use duration were excluded from analysis; ^b^coefficient; ^c^odds ratio; ^d^infants born before 37 weeks gestational age were excluded from this analysis.

*p < 0.05.

## 4 Discussion

In this assessment of infant outcomes following PrEP programmatic implementation in Kenya, we found that maternal PrEP use during pregnancy was not associated with meaningful differences in preterm birth, gestational age, birthweight, congenital anomalies or infant growth at six weeks among the general population or among AGYW mothers. While the results suggest a possible protective effect of PrEP use on preterm birth and LBW, these results are difficult to interpret given small numbers in the exposed group and wide confidence intervals. While the PrEP unexposed group had a later average gestational age at PrEP screening (25 vs. 28 weeks), both groups had similar mean gestational age at birth (38.5 weeks). Additionally, the majority of women who initiated PrEP did so on the same day as their initial PrEP screening. The PrIYA program was not designed to include a delay between PrEP screening and initiation, although women were able to return on later dates to initiate PrEP.

These results are similar to PrEP safety studies among women who became pregnant during the efficacy trials or demonstration projects 14, 16. In an analysis of pregnancies in the Partners PrEP Study, PrEP exposure was discontinued when the pregnancy was identified (on average at six weeks’ gestation), and one‐month infant growth outcomes were not associated with PrEP exposure 14. Heffron *et al*. recently analysed infant growth outcomes among 30 PrEP exposed and 96 unexposed infants in the Partners Demonstration Project 16. This analysis found no impact on preterm birth or weight one‐month postpartum; however, the analysis was limited by the small number of PrEP exposed infants. Our analysis in a larger cohort of prenatal PrEP users found similar results on preterm birth and six week growth outcomes.

Current recommendations and safety evidence for the use of PrEP in pregnancy are based largely on safety evidence from TDF‐based ART regimens in pregnancy. Studies among HIV‐exposed, uninfected infants have shown no statistical differences in infant growth outcomes at six weeks postpartum with TDF‐based ART exposure compared with infants with no TDF exposure 10. However, studies which assessed infant growth at later time points have demonstrated conflicting results on the impact of *in utero* TDF‐based ART exposure on infant outcomes 10, 19. While we did not find evidence of growth compromise following prenatal PrEP exposure, it is possible that the six week time frame of this analysis is not long enough to assess the impact of PrEP on infant growth. Future analyses should consider collecting infant growth data at later time points to better define the impact of *in utero* TDF exposure on infant growth.

This analysis has several limitations. We were likely not able to identify all women who use PrEP in pregnancy or who were screened for PrEP by PrIYA staff. PrIYA staff used all available resources (self‐report and clinical records) to identify women who were screened for or used PrEP in pregnancy. There is the possibility of recall bias, however, given the increased documentation of PrEP initiation and refill visits in the MCH card, it is more likely that women who were screened for PrEP but did not initiate were not included in this analysis. Recall bias regarding taking PrEP was minimized by confirming PrEP screening and exposure in medical records. We relied on self‐report and medical records of PrEP dispensation to define PrEP exposure rather than biological assessment of PrEP adherence with drug levels. This may have resulted in women who never actually took PrEP being included in the exposed group, thus diluting any potential impact of PrEP. While we did ask women who reported receiving PrEP if they ever swallowed the bills, there is a significant likelihood of women inaccurately reporting taking PrEP. An ongoing trial will assess infant outcomes among PrEP exposed and unexposed infants and address many of these limitations 32.

Since birth and infant growth outcomes were assessed among mothers with live infants who attended postnatal services, these results are biased by the low risk nature of the population of mothers and infants attending the standard six week postnatal care visits. As such, we were unable to assess the impact of PrEP exposure on miscarriage, stillbirth, neonatal mortality or infants that are hospitalized due to preterm birth or other complications at six weeks postpartum. These results may not include potential critical negative impacts of PrEP use in pregnancy on perinatal mortality. Finally, we were not able to assess the accuracy of the clinical records abstracted, in particular, we did not assess the five infants with reported congenital malformations for additional information on the type or severity of these malformations. Since these five cases were in PrEP unexposed infants, PrEP exposure was not a contributing factor.

These results provide additional, programmatic, evidence that short‐term infant outcomes were not adversely affected by prenatal PrEP use in the general population and specifically among AGYW mothers. PrIYA has demonstrated that monitoring of infant outcomes is feasible within large‐scale programmatic implementation of PrEP with at risk pregnant and postpartum populations. Given the WHO call for continued safety monitoring of PrEP in at‐risk pregnant and postpartum women, as countries and programmes work to incorporate PrEP into clinical settings, other programmes should consider collecting similar data to monitor the safety of PrEP in this population.

## 5 Conclusions

This is the largest analysis to date of birth and early infant growth outcomes among PrEP exposed and unexposed infants which provides additional evidence that prenatal PrEP does not have adverse effects on infant outcomes. As PrEP use expands among pregnant and postpartum women, it is critical for other programmes to consider collecting and assessing outcomes among PrEP exposed and unexposed infants.

## Competing interests

The authors have no competing interests to declare**.**

## Authors’ contributions

GJS, JMB, JK, KM, JP designed the PrIYA intervention. GJS and JMB are Multiple Principal Investigators. JK is the Country PI, KM and JP are Project Directors. JCD and EB developed the statistical analysis plan. EB conducted the statistical analyses. GJS, JMB, JK, JP, and KM provided scientific expertise. FA, HL, JS, oversee data collection and data management activities. JCD, JP, KM, FA, HL, and JS oversee implementation of study procedures. All authors have contributed to the development of this manuscript, have read and approved the final version for publication.

## Supporting information


**Table S1.** Birth and infant growth outcomes by prenatal PrEP exposure among mothers aged 15 to 24
**Table S2.** Birth and infant growth outcomes by trimester of PrEP initiation among AGYW (15 to 25 years old)
**Table S3.** Birth and infant growth outcomes by duration of PrEP use among mothers aged 15 to 24
