# Supplementary material for: Perinatal outcomes following maternal pre‐exposure prophylaxis (PrEP) use during pregnancy: results from a large PrEP implementation program in Kenya
Source: J Int AIDS Soc. 2019 Sep 9;22(9):e25378. doi: 10.1002/jia2.25378 (PMC6733245; doi:10.1002/jia2.25378)
Supplement: Supplementary file 1 — Table S1. Birth and infant growth outcomes by prenatal PrEP exposure among mothers aged 15 to 24 Table S2. Birth and infant growth outcomes by trimester of PrEP initiation among AGYW (15 to 25 years old) Table S3. Birth and infant growth outcomes by duration of PrEP use among mothers aged 15 to 24 [file JIA2-22-e25378-s001.docx]

| **Table S1: Birth and infant growth outcomes by prenatal PrEP exposure among mothers aged 15- 24** | | | | | | | | |
| --- | --- | --- | --- | --- | --- | --- | --- | --- |
|  | **Overall**  **(N=775)** | | **PrEP Exposed**  **(N=113)** | | **PrEP Unexposed**  **(N=662)** | | **Unadjusted**  **Coeff. or OR**  **(95% CI)** | **Adjusted**  **Coeff. or OR**  **(95% CI)** |
|  | **Mean or N (%)** | **95% CI** | **Mean or N (%)** | **95% CI** | **Mean or N (%)** | **95% CI** |  |  |
| **Birth Outcomes** | | | | | | | | |
| Gestational age at birth (weeks)^a^ | 38.5 | 38.2, 38.8 | 38.5 | 38.0, 38.9 | 38.5 | 38.2, 38.8 | -0.055  (-0.43, 0.32) | 0.18  (-0.27, 0.62) |
| Preterm birth^b^ | 47 (6.1) | 2.7, 3.2 | 4 (3.5) | 1.7, 7.2 | 43 (6.5) | 2.6, 15.2 | 0.53  (0.18, 1.51) | 0.35  (0.13, 0.95)* |
| Congenital malformation^b^ | 2 (0.3) | 0.1, 1.0 | 0 (0) | -- | 2 (0.3) | 0.1, 1.2 | -- | -- |
| Birth weight (kg)^a^ | 3.3 | 3.2, 3.3 | 3.3 | 3.2, 3.5 | 3.3 | 3.2, 3.3 | 0.083  (-0.042, 0.21) | 0.19  (0.080, 0.31)* |
| Low birth weight^b^ | 18 (2.5) | 1.4, 4.3 | 1 (0.9) | 0.1, 8.4 | 17 (2.7) | 1.6, 4.8 | 0.67  (0.15, 3.07) | 0.31  (0.032, 2.96) |
| **6-Week Infant Growth Outcomes** | | | | | | | |  |
| Weight (kg)^a^ | 5.0 | 4.9, 5.1 | 4.9 | 4.8, 5.1 | 5.0 | 4.9, 5.2 | -0.11  (-0.24, 0.018) | -0.083  (-0.28, 0.12) |
| Absolute weight-for-age z-score (WAZ)^a^ | 0.3 | 0.2, 0.5 | 0.2 | 0.02, 0.5 | 0.4 | 0.2, 0.5 | -0.11  (-0.32, 0.10) | -0.055  (-0.33, 0.22) |
| Moderate-to-severe underweight^b^ | 16 (2.5) | 1.2, 5.3 | 3 (3.4) | 1.3, 8.7 | 13 (2.3) | 1.0, 5.4 | 1.47  (0.61, 3.56) | 1.44  (0.37, 5.64) |
| Length (cm)^a^ | 55.5 | 54.9, 56.2 | 55.3 | 54.7, 56.0 | 55.6 | 54.8, 56.3 | -0.25  (-1.04, 0.55) | -0.48  (-1.45, 0.49) |
| Absolute length-for-age z-score (LAZ)^a^ | -0.1 | -0.4, 0.3 | -0.1 | -0.5, 0.2 | -0.1 | -0.4, 0.3 | -0.091  (-0.50, 0.32) | -0.22  (-0.73, 0.30) |
| Moderate-to-severe stunting^b^ | 62 (9.9) | 6.8, 14.2 | 11 (12.6) | 6.6, 22.7 | 51 (9.4) | 5.8, 14.9 | 1.39  (0.58, 3.36) | 2.61  (0.73, 9.40) |
| Weight-for-length (WHZ) Z-score^a^ | 0.7 | 0.3, 1.0 | 0.7 | 0.2, 1.2 | 0.7 | 0.3, 1.1 | 0.05  (-0.44, 0.54) | 0.25  (-0.23, 0.72) |
| Moderate-to-severe wasting^b^ | 43 (7.0) | 4.2, 11.5 | 6 (7.1) | 2.7, 17.0 | 37 (7.0) | 4.0, 12.0 | 1.01  (0.39, 2.65) | 0.93  (0.18, 4.92) |
| ^a^ Coefficient  ^b^ Odds Ratio  *P-value <0.05  Adjusted for gestational age at PrEP screening and Partner HIV status | | | | | | | | |

| **Table S2: Birth and infant growth outcomes by trimester of PrEP initiation among AGYW (15-25 years old)** | | | | | | | | | | | | | | |
| --- | --- | --- | --- | --- | --- | --- | --- | --- | --- | --- | --- | --- | --- | --- |
|  | **Unexposed** | | **Trimester of PrEP initiation^1^** | | | | | | | | | | | |
|  | **Reference**  (n=542) | | **First**  (n=6) | | | | **Second**  (n=43) | | | | **Third**  (n=38) | | | |
|  | Mean  N (%) | 95% CI | Mean  N (%) | 95% CI | Coeff. or OR  (95% CI) | Adjusted Coeff. or OR  (95% CI) | Mean  N (%) | 95% CI | Coeff. or OR  (95% CI) | Adjusted Coeff. or OR  (95% CI) | Mean  N (%) | 95% CI | Coeff. or OR  (95% CI) | Adjusted Coeff. or OR  (95% CI) |
| Birth Outcomes | | | | | | | | | | | | | | |
| Gestational age at birth (weeks)^3^ | 38.5 | 38.2, 38.8 | 38.8 | 38.1, 39.5 | 0.26  (-0.37, 0.90) | 0.25  (-0.37, 0.87) | 38.5 | 37.9, 39.0 | -0.061  (-0.57, 0.45) | -0.065  (-0.58, 0.45) | 38.6 | 37.9, 39.3 | 0.068  (-0.50, 0.64) | 0.062  (-0.51, 0.63) |
| Preterm birth^4^ | 43 (6.5) | 2.6, 15.2 | 0 (0.0) | -- | -- | -- | 3 (5.5) | 2.0, 13.8 | 0.83 (0.24, 2.89) | 0.87 (0.28, 2.76) | 0 (0.0) | -- | -- | -- |
| Congenital malformation^4^ | 2 (0.3) | 0.1, 1.2 | 0 (0.0) | -- | -- | -- | 0 (0.0) | -- | -- | -- | 0 (0.0) | -- | -- | -- |
| Birth weight (kg)^3^ | 3.3 | 3.2, 3.3 | 3.2 | 2.8, 3.7 | -0.022  (-0.41, 0.37) | 0.0041  (-0.38, 0.38) | 3.3 | 3.1, 3.4 | 0.02  (-0.10, 0.14) | 0.032  (-0.091, 0.15) | 3.5 | 3.3, 3.7 | 0.21  (0.012, 0.41) | 0.23  (0.031, 0.42) |
| Low birth weight  (<2.5 kg)^4,5^ | 17 (2.7) | 1.6, 4.8 | 0 (0.0) | -- | -- | -- | 1 (1.9) | 0.2, 15.3 | 0.69  (0.087, 5.51) | 0.64  (0.086, 4.81) | 0 (0.0) | -- | -- | -- |
| Infant Growth Outcomes at 6 Weeks Postpartum | | | | | | | | | | | | | | |
| Weight (kg)^3^ | 5.0 | 4.9, 5.2 | 4.8 | 4.2, 5.5 | -0.22  (-0.79, 0.36) | -0.19  (-0.75, 0.38) | 4.8 | 4.6, 5.0 | -0.27*  (-0.44,  -0.10) | -0.26*  (-0.44,  -0.08) | 5.1 | 4.9, 5.3 | 0.075  (-0.15, 0.30) | 0.089  (-0.16, 0.34) |
| Absolute WAZ^3^ | 0.4 | 0.2, 0.5 | -0.04 | -1.2, 1.2 | -0.40  (-1.36, 0.56) | -0.34  (-1.28, 0.60) | 0.1 | -0.3, 0.5 | -0.25  (-0.61, 0.11) | -0.23  (-0.61, 0.15) | 0.4 | 0.1, 0.8 | 0.077  (-0.28, 0.43) | 0.11  (-0.28, 0.49) |
| Moderate-to-severe underweight^4^ | 13 (2.3) | 1.0, 5.4 | 0 (0.0) | -- | -- | -- | 2 (4.8) | 1.1, 18.8 | 2.08  (0.63, 6.91) | 1.90  (0.63, 5.73) | 1 (2.5) | 0.4, 13.3 | 1.07  (0.18, 6.42) | 0.92  (0.16, 5.45) |
| Length (cm)^3^ | 55.6 | 54.8, 56.3 | 57.4 | 53.8, 61.1 | 1.86  (-1.23, 4.95) | 2.02  (-1.30, 5.34) | 54.5 | 53.6, 55.4 | -1.06*  (-2.01,  -0.12) | -1.01*  (-2.00,  -0.29) | 55.9 | 54.6, 57.2 | 0.34  (-0.70, 1.39) | 0.42  (-0.79, 1.63) |
| Absolute LAZ^3^ | -0.06 | -0.4, 0.3 | 0.8 | -1.2, 2.8 | 0.85  (-0.86, 2.57) | 0.94  (-0.91, 2.79) | -0.5 | -0.9, -0.1 | -0.44  (-0.93, 0.038) | -0.42  (-0.92, 0.087) | 0.1 | -0.5, 0.7 | 0.16  (-0.38, 0.69) | 0.20  (-0.42, 0.83) |
| Moderate-to-severe stunting^4^ | 51 (9.4) | 5.8, 14.9 | 0 (0.0) | -- | -- | -- | 8 (18.6) | 8.0, 37.4 | 2.20  (0.76, 6.36) | 2.04 (0.70, 5.99) | 3 (7.9) | 3.2, 18.1 | 0.82  (0.32, 2.09) | 0.72  (0.27, 1.96) |
| Weight-for-length (WHZ) z-score^3^ | 0.7 | 0.3, 1.1 | 0.2 | -3.1, 3.4 | -0.50  (-2.78, 1.78) | -0.46  (-2.79, 1.87) | 0.8 | 0.1, 1.4 | 0.12  (-0.52, 0.76) | 0.13  (-0.56, 0.83) | 0.7 | 0.2, 1.3 | 0.049  (-0.43, 0.53) | 0.072  (-0.49, 0.63) |
| Moderate-to-severe wasting^4^ | 37 (7.0) | 4.0, 12.0 | 1 (20.0) | 0.8, 88.9 | 3.32  (0.34, 32.24) | 3.73  (0.35, 40.1) | 4 (9.5) | 2.9, 26.9 | 1.40  (0.39, 4.96) | 1.46 (0.41, 5.20) | 1 (2.6) | 0.2, 22.9 | 0.36 (0.057, 2.27) | 0.38  (0.051, 2.85) |
| ^1^ Participant without trimester of PrEP initiation data excluded from analysis  ^2^ 17 participants without information on PrEP use duration were excluded from analysis  ^3^ Coefficient  ^4^ Odds Ratio  ^5^ Infants born before 37 weeks gestational age were excluded from this analysis  *p-value <0.05  Adjusted for partner HIV status | | | | | | | | | | |  |  |  |  |

| **Table S3: Birth and infant growth outcomes by duration of PrEP use among mothers aged 15- 24** | | | | | | | | | | |
| --- | --- | --- | --- | --- | --- | --- | --- | --- | --- | --- |
|  | **Unexposed** | | **PrEP duration^2^** | | | | | | | |
|  | **Reference**  (n=662) | | **Short Duration**  (n=47) | | | | **Long Duration**  (n=57) | | | |
|  | Mean  N (%) | 95% CI | Mean  N (%) | 95% CI | Coeff. or OR  (95% CI) | Adjusted Coeff. or OR  (95% CI) | Mean  N (%) | 95% CI | Coeff. or OR  (95% CI) | Adjusted Coeff. or OR  (95% CI) |
| Birth Outcomes | | | | | | | | | | |
| Gestational age at birth (weeks)^3^ | 38.5 | 38.2, 38.8 | 38.4 | 37.8, 39.1 | -0.068  (-0.68, 0.55) | -0.074  (-0.69, 0.54) | 38.6 | 38.0, 39.2 | 0.081  (-0.40, 0.56) | 0.075  (-0.41, 0.56) |
| Preterm birth^4^ | 43 (6.5) | 2.6, 15.2 | 2 (4.3) | 1.2, 13.6 | 0.64  (0.14, 2.94) | 0.69  (0.16, 3.00) | 1 (1.8) | 0.1, 14.2 | 0.26  (0.039, 1.70) | 0.28  (0.051, 1.55) |
| Congenital malformation^4^ | 2 (0.3) | 0.1, 1.2 | 0 (0.0) | --- | -- | -- | 0 (0.0) | -- | -- | -- |
| Birth weight (kg)^3^ | 3.3 | 3.2, 3.3 | 3.5 | 3.3, 3.7 | 0.21*  (0.040, 0.39) | 0.23*  (0.060, 0.39) | 3.2 | 3.1, 3.4 | -0.022  (-0.17, 0.12) | -0.0071  (-0.15, 0.14) |
| Low birth weight  (<2.5 kg)^4,5^ | 17 (2.7) | 1.6, 4.8 | 0 (0.0) | -- | -- | -- | 1 (1.8) | 0.2, 15.4 | 0.64  (0.076, 5.47) | 0.59  (0.072, 4.66) |
| Infant Growth Outcomes at 6 Weeks Postpartum | | | | | | | | | | |
| Weight (kg)^3^ | 5.0 | 4.9, 5.2 | 5.1 | 4.8, 5.3 | 0.037  (-0.20, 0.28) | 0.044  (-0.22, 0.31) | 4.8 | 4.6, 5.0 | -0.25  (-0.44, 0.064) | -0.24  (-0.44, 0.035) |
| Absolute WAZ^3^ | 0.4 | 0.2, 0.6 | 0.5 | 0.2, 0.8 | 0.13  (-0.22, 0.47) | 0.15  (-0.22, 0.52) | 0.2 | -0.03, 0.5 | -0.31  (-0.64, 0.011) | -0.28  (-0.64, 0.074) |
| Moderate-to-severe underweight^4^ | 13 (2.3) | 1.0, 5.4 | 0 (0.0) | -- | -- | -- | 3 (6.5) | 2.6, 15.6 | 2.91  (1.19, 7.10)* | 2.48  (1.15, 5.37)* |
| Length (cm)^3^ | 55.6 | 54.8, 56.3 | 55.7 | 54.5, 56.9 | 0.13  (-0.85, 1.11) | 0.19  (-0.94, 1.31) | 55.3 | 54.5, 56.0 | -0.32  (-1.33, 0.70) | -0.23  (-1.41, 0.95) |
| Absolute LAZ^3^ | -0.6 | -0.4, 0.3 | -0.03 | -0.7, 0.6 | 0.032  (-0.48, 0.55) | 0.066  (-0.53, 0.66) | -0.1 | -0.5, 0.2 | -0.070  (-0.58, 0.45) | -0.017  (-0.62, 0.59) |
| Moderate-to-severe stunting^4^ | 51 (9.4) | 5.8, 14.9 | 4 (11.8) | 4.2, 28.8 | 1.28  (0.41, 3.96) | 1.14  (0.35, 3.78) | 5 (10.9) | 5.3, 21.0 | 1.17  (0.45, 3.05) | 0.99  (0.37, 2.64) |
| Weight-for-length (WHZ) z-score^3^ | 0.7 | 0.3, 1.1 | 0.8 | 0.3, 1.7 | 0.18  (-0.49, 0.84) | 0.19  (-0.56, 0.94) | 0.5 | -0.2, 1.1 | -0.21  (-0.84, 0.41) | -0.19  (-0.90, 0.51) |
| Moderate-to-severe wasting^4^ | 37 (7.0) | 4.0, 12.0 | 2 (6.1) | 1.4, 23.1 | 0.86  (0.25, 2.97) | 0.91  (0.23, 3.64) | 4 (8.9) | 3.2, 22.4 | 1.30  (0.43, 3.93) | 1.41  (0.46, 4.34) |
| ^1^ Participant without trimester of PrEP initiation data excluded from analysis  ^2^ 17 participants without information on PrEP use duration were excluded from analysis  ^3^ Coefficient  ^4^ Odds Ratio  ^5^ Infants born before 37 weeks gestational age were excluded from this analysis  *p-value <0.05  Adjusted for partner HIV status | | | | | | | | | | |
